# The influence of high glucose conditions on macrophages and its effect on the autophagy pathway

**DOI:** 10.3389/fimmu.2023.1130662

**Published:** 2023-04-12

**Authors:** Emanuella S. A. Sousa, Luiz A. D. Queiroz, João P. T. Guimarães, Kamilla C. Pantoja, Rafael S. Barros, Sabrina Epiphanio, Joilson O. Martins

**Affiliations:** ^1^ Laboratory of Immunoendocrinology, School of Pharmaceutical Sciences, Department of Clinical and Toxicological Analyses, University of São Paulo, São Paulo, Brazil; ^2^ Laboratory of Malaria Cellular and Molecular Immunopathology, School of Pharmaceutical Sciences, Department of Clinical and Toxicological Analyses, University of São Paulo, São Paulo, Brazil

**Keywords:** inflammation, diabetes mellitus, hyperglycemia, autophagy, macrophages

## Abstract

**Introduction:**

Macrophages are central cells in mediating the inflammatory response.

**Objective and Methods:**

We evaluated the effect of high glucose conditions on the inflammatory profile and the autophagy pathway in Bone-Marrow Derived Macrophages (BMDM) from diabetic (D-BMDM) (alloxan: 60mg/kg, i.v.) and non-diabetic (ND-BMDM) C57BL/6 mice. BMDM were cultured in medium with normal glucose (5.5 mM), or high glucose (25 mM) concentration and were primed with Nigericin (20µM) stimulated with LPS (100 ng/mL) at times of 30 minutes; 2; 4; 6 and 24 hours, with the measurement of IL-6, IL-1β and TNF-α cytokines.

**Results:**

We have further identified changes in the secretion of pro-inflammatory cytokines IL-6, IL-1β and TNF-α, where BMDM showed increased secretion of these cytokines after LPS + Nigericin stimulation. In addition, changes were observed in the autophagy pathway, where the increase in the autophagic protein LC3b and Beclin-1 occurred by macrophages of non-diabetic animals in hyperglycemic medium, without LPS stimulation. D-BMDM showed a reduction on the expression of LC3b and Beclin-1, suggesting an impaired autophagic process in these cells.

**Conclusion:**

The results suggest that hyperglycemia alters the inflammatory pathways in macrophages stimulated by LPS, playing an important role in the inflammatory response of diabetic individuals.

## 1 Introduction

Macrophages are innate immune cells, which plays a central role in modulating the inflammatory response against infections (1). These cells can polarize depending on the stimuli they receive, being classified into classic activated (M1), and alternatively activated (M2) macrophages (2).

The classic pathway can be activated by bacterial components like lipopolysaccharides (LPS), IFN-γ and granulocyte macrophage colony-stimulating factor (GM-CSF), assuming a pro-inflammatory phenotype, increasing the secretion of cytokines such as Tumor Necrosis Factor alpha (TNF-α), Interleukin 1 beta (IL-1β), IL-6, IL-12, IL-18, stimulating its antimicrobial activity by producing reactive oxygen species (ROS), nitric oxide (NO), and antimicrobial peptides (3, 4). On the other hand, the alternative phenotype, or M2, is activated in response to the cytokines IL-4 and IL-13, promoting an anti-inflammatory response by expressing high levels of IL-10 and transforming growth factor (TGF-β), starting tissue repair (5).

Besides its capacity to induce M1 polarization, LPS plays a role on activating other pathways in bone marrow-derived macrophages (BMDM) such as the autophagy pathway, by its binding to the Toll-Like Receptor 4, enhancing the phagocytic process in these cells (6). Autophagy is a regulatory process that preserves cell homeostasis, degrading and recycling cytoplasmic components in response to environmental variations, starvation, infections, and stress to maintain a healthy and functional intracellular environment (7). This process begins with the formation of the autophagosome, a double-membrane structure that surrounds the cytoplasmic cargo (macromolecules and organelles) and then fuses with the lysosome, resulting in the degradation of the cargo (8). Several proteins involved in the biogenesis of endosomes and phagosomes participate in the regulation of autophagy, and a wide range of proteins from autophagy-related genes (ATG), including ATG8 (LC3), which together with Beclin-1, a protein that acts during the initial autophagic process, it is commonly used as a marker to assess the autophagosome (9, 10). The role of autophagy in the regulation of inflammation during infectious and non-infectious diseases is already well established (11, 12), being described that the negative regulation of this process interferes in the inflammatory responses to diseases (13, 14).

It is known that Nigericin, an ionophore derived from *Streptomyces Hygroscopicus* combined with LPS induces the secretion of IL-1β through the induction of NLRP3 inflammasome, a component of the innate immune system (15–17). Besides being known as a survival mechanism for cells under stress conditions, studies indicates that autophagy may contribute to cell death processes under pathological conditions, increasing the inflammatory response (18–20), being known that a failure on the autophagy machinery can lead to an increase on IL-1β secretion (21). This pro-inflammatory cytokine is central on the inflammatory response, where a sustained secretion of IL-1β can drives to a persistent inflammatory state and consequent tissue injury (22, 23).

Macrophages are influenced by hyperglycemia (18, 24). Long-term hyperglycemic states sensitize macrophages to increase the secretion of cytokines, reducing their phagocytic activity and NO production (19, 25), and BMDM from diabetic mice showed an increased proinflammatory gene expression, even when cultured in normal glucose conditions, suggesting a priming effect driven by hyperglycemia (20, 26). Type 1 diabetes mellitus (T1DM) is a disease caused by the autoimmune destruction of insulin-producing beta cells, which directly interferes in glucose metabolism, leading to a hyperglycemic state. The hyperglycemia presented in diabetic individuals has several deleterious effects, which may occur due to the activation of pathways that stimulate ROS production, which in the absence of an appropriate antioxidant defense mechanism, promotes the activation of intracellular stress-sensitive pathways, causing damage to cells and consequent development of complications related to the pathogenesis of the disease (21–23, 27–29).

Thus, in this study, we investigated the influence of hyperglycemia on the inflammatory response *in vitro* of macrophages stimulated with LPS and nigericin, in a hyperglycemic environment, and its effect on the autophagy process.

## 2 Material and methods

### 2.1 Animals

In this study, we used 8–12-week-old C57BL/6 mice, weighing approximately 25–30 g. The animals were maintained at 23 ± 2 °C, in mini-isolators, with a maximum of 3 mice, in a 12-hour light/dark cycle, with water and food available, undergoing an acclimation period of 7 days before diabetes induction. Artificial devices were placed in the cages to enrich the environment (cotton, igloo, cardboard tubes etc.).

### 2.2 Diabetes induction

For the T1DM model, 60 mg/kg of alloxan (Sigma-Aldrich, St. Louis, Missouri, USA) was administered intravenously, with saline solution administered. On non-diabetic animals, only saline solution was administered (24, 30). On day 0, blood glucose was measured using a glucometer Accu-Chek Advantage II (Roche Diagnostics, São Paulo, SP, Brazil) and the animals where weighed. After 10 days of induction, weight and blood glucose were measured again to confirm the induction of diabetes. This study was accepted by the Ethics Committee on Animal Use (CEUA) at the School of Pharmaceutical Sciences (FCF), the University of São Paulo, Brazil (protocol number: CEUA/FCF/570), and all experiments were conducted in strict accordance with the principles and guidelines of the National Council for the Control of Animal Experimentation (CONCEA).

### 2.3 Bone marrow derived macrophages and cell culture

First, the femurs were collected its ends were cut, and BMDM were collected by washing the inside of the bones with sterile PBS and were incubated for differentiation into BMDM with R20/30 medium: 50% of RPMI-1640 medium (Gibco, Gaithersburg, MD, USA Sigma-Aldrich), 20% fetal bovine serum (FBS) (Sigma-Aldrich, St. Louis, Missouri, USA) and 30% of L929 cell conditioned medium (LCCM) for seven days, where on the fourth day, a fresh medium was added (25, 31). After seven days, macrophages were collected using ice-cold PBS, centrifuged at 1500 rpm for 5 minutes at 4°C, and resuspended in RPMI-1640 medium (Gibco^®^ by Life Technologies, Thermo Fisher Scientific, Waltham, Massachusetts, EUA), LCCM and FBS (85% RPMI-1640 medium, 5% LCCM and 10% FBS). Cell counts were performed with trypan blue (Gibco^®^ by Life Technologies, Thermo Fisher Scientific, Waltham, Massachusetts, USA). The final volume of the suspension, containing 1x10^6^ cells per mL, was distributed into four wells (2mL per well, totalizing 2x10^6^ cells per well) and incubated at 37°C, 5% CO2 for adhesion. After 12 hours, cells were primed with LPS (Sigma-Aldrich, Saint Louis, Missouri, EUA) (100ng/mL) for 4 hours and Nigericin (Sigma-Aldrich, Saint Louis, Missouri, EUA) (20 µM) for 30 minutes. After priming, cells were cultured at a standard concentration (5.5 mM) and a high concentration of glucose (Gibco^®^ by Life Technologies, ThermoFisher Scientific, Waltham, Massachusetts, EUA) (25 mM) and stimulated with LPS at a concentration of 100 ng/mL (24, 26, 30, 32).

### 2.4 Cytokines measurements

To measure the serum concentrations of TNF-α, IL-6 and IL-1β, in the culture supernatants obtained after 30 minutes, 2;4;6 and 24 hours of incubation, Enzyme-linked immune assays (ELISA) were performed following the manufacturer’s protocols (R&D^®^ Systems, Inc., Minneapolis, MN, USA).

### 2.5 Western blot assay

Macrophages were cultured in 6-well plates at a density of 2 × 10^6^ and incubated for 30 minutes. We used RIPA lysis buffer to lysate the BMDM. The protein extract was measured using a Pierce™ BCA Protein Assay kit (Thermo Fisher Scientific, Waltham, Massachusetts, USA). We used 20 µg of protein extract and separated in a polyacrylamide gel (10-15%). For protein transfer to the nitrocellulose membrane we used a semi-dry system using transfer buffer (25 mM Tris; 192 mM glycine; 0.1% SDS; 20% methanol) at 60 mA for 60 minutes. After transfer, the membranes were blocked using 5% bovine serum albumin (BSA) in Tris-buffered saline containing Tween-20 (TBST) for 1 hour. Next, the membranes were incubated with a primary antibody overnight in a 5% BSA solution in TBST at 4 °C. After incubation, membranes were washed three times with TBST for 10 minutes each, and incubated with a secondary antibody for 1 hour and then washed three times with TBST for 10 minutes each. The revelation was performed using ECL (enhanced chemiluminescence - ECL; Amersham, Arlington Heights, IL) on the Amersham Imager 680 blot and gel imager equipment (Amersham, Buckinghamshire, United Kingdom).

The primary anti-mouse LC3B and anti-mouse Beclin-1 antibodies purchased from Cell Signaling Technology (Cell Signaling Technology^®^, Danvers, Massachusetts, USA) were used to detect the target proteins. We used an HRP-conjugated goat anti-rabbit IgG H&L antibody (Abcam) as secondary antibodies. Relative band densities were determined by densitometric analysis using ImageJ software. Band density values ​​were normalized with β-actin anti-mouse antibody (Cell Signaling Technology^®^, Danvers, Massachusetts, USA).

### 2.6 Statistical analysis

The results were evaluated by analysis of variance (two-way ANOVA) followed by the Tukey-Kramer multiple comparisons test using GraphPad software 9.0 (San Diego, CA, USA). The data are presented as the standard error of the mean (SEM), with p < 0.05 considered to represent a significant difference.

## 3 Results

### 3.1 Alloxan T1D model establishment

**Figure 1:**
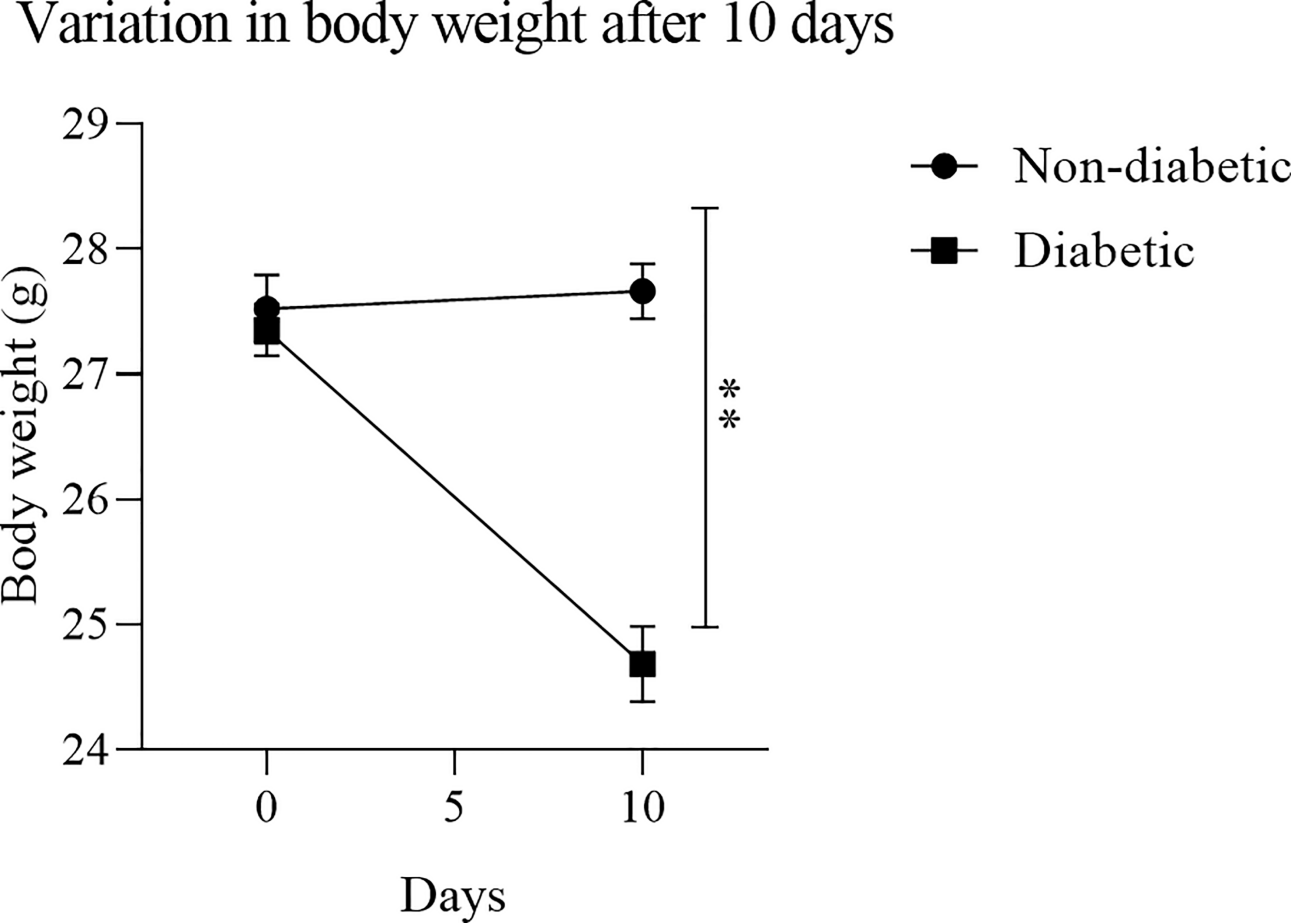
Variation in body mass after diabetes induction: After treatment with the diabetogenic agent Alloxan (60mg/kg), it was observed that the induced animals had a significant weight loss. Results expressed in Sidak Simultaneous Comparison Test **p<0.005, n=13.

**Figure 2:**
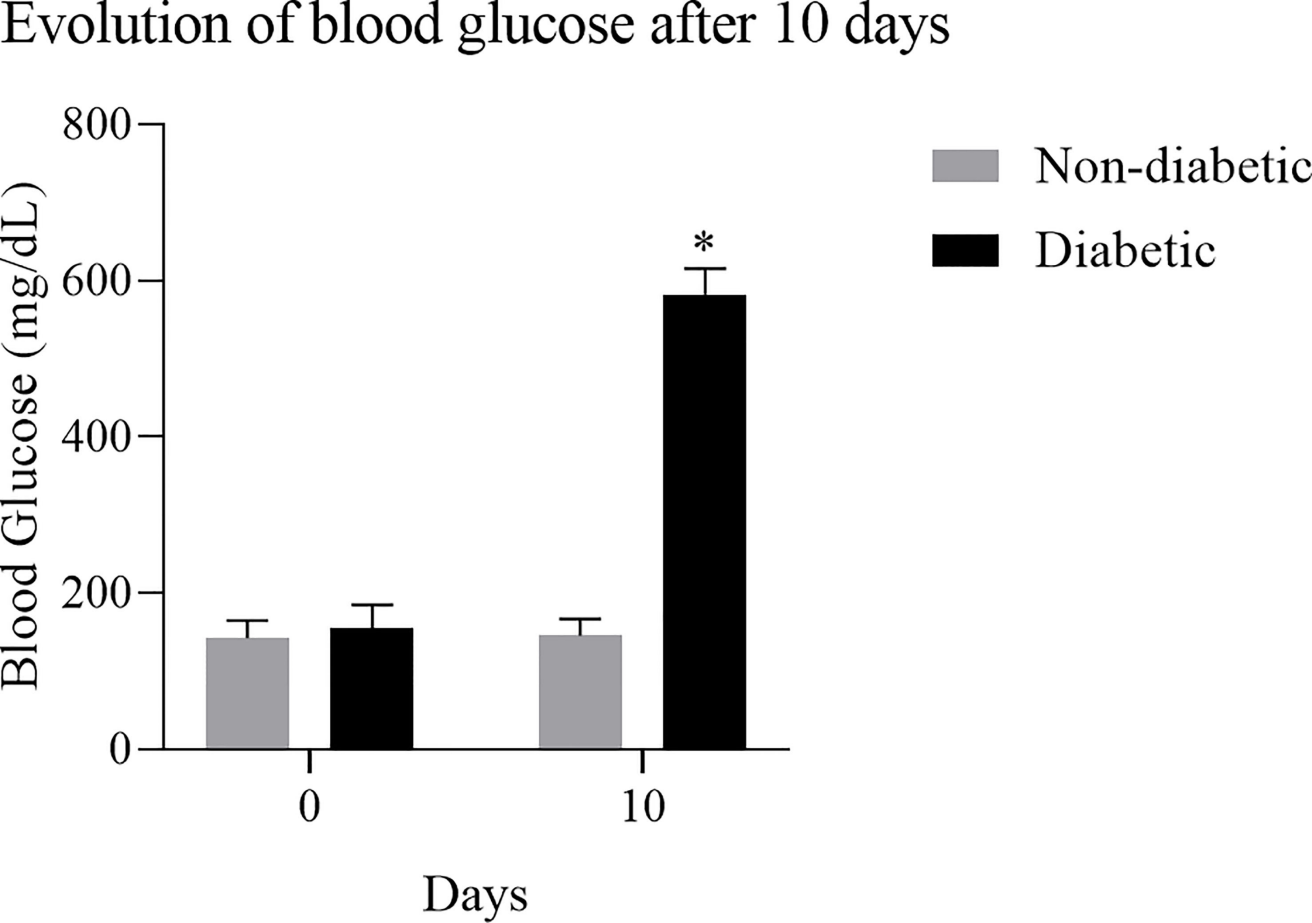
Evolution of blood glucose after induction of diabetes: Diabetic animals showed an increase in blood glucose after 10 days of induction with the diabetogenic agent. Results expressed in Sidak Simultaneous Comparison Test *p<0.005, n=13.

To obtain BMDM from a hyperglycemic environment, we used an alloxan T1DM animal model. After 10 days of induction with alloxan, when compared to the control group, the animals showed a loss of body weight (**Figure 1**), and an increase in blood glucose (**Figure 2**).

### 3.2 The involvement of hyperglycemia in the autophagy process in BMDM of diabetic animals stimulated with LPS+Nigericin

**Figure 3:**
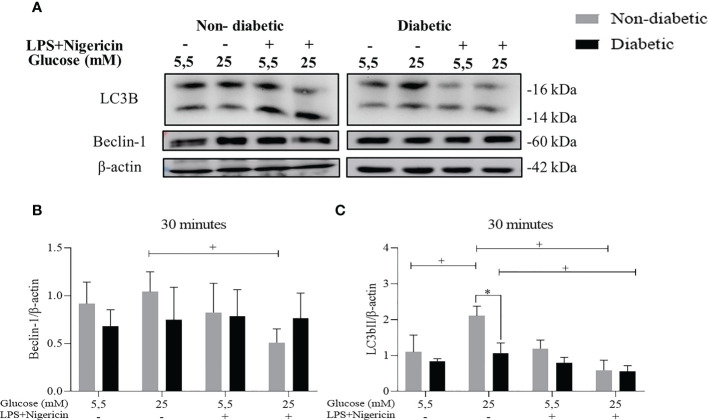
Evaluation of the autophagy pathway. **(A)** Expression **(B)** Beclin-1/β-actin protein level within 30 minutes. +P<0.05 ND-BMDM 25mM- *vs* ND-BMDM 25mM+; **(C)** LC3b/β-actin protein level after 30 minutes of treatment. +P<0.05 ND-BMDM 5.5mM- *vs* ND-BMDM 25mM-; +P<0.05 ND-BMDM 25mM- *vs* ND-BMDM 25mM+; +P<0.05 D-BMDM 25mM- *vs* D-BMDM 25mM+; *P<0.05 ND-BMDM 25mM- *vs* D-BMDM 25mM-. Results represent mean ± SEM. N = 3-6. BMDM: Bone-Marrow Derived Macrophages, from diabetic (D-) and non-diabetic (ND-) C57BL/6 mice.

To assess the autophagy pathway in BMDM, the protein expression of LC3B and Beclin-1 were evaluated by the Western Blotting method, after 30 minutes (**Figure 3A**). We identified that ND-BMDM had an increase in Beclin-1 expression in hyperglycemic medium without stimulation (**Figure 3B**). Furthermore, when the LC3b protein was evaluated, we observed an increase in the expression of this protein by ND-BMDM and D-BMDM in HG medium without LPS+Nigericin stimulation (**Figure 3C**).

### 3.3 IL-6 cytokine secretion in BMDM of diabetic animals stimulated with LPS and Nigericin

**Figure 4:**
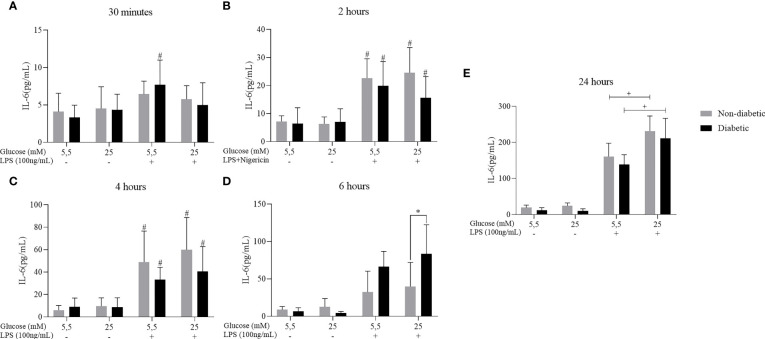
IL-6 secretion by ND-BMDM and D-BMDM with Nigericin priming and LPS stimulation. IL-6 release after **(A)** 30 minutes with different glucose concentrations. #P<0.05 D-BMDM 5.5Mm+ *vs* D-BMDM 5.5mM-. **(B)** 2 hours with different glucose concentrations. #P<0.05 stimulated groups *vs* non stimulated. **(C)** 4 hours with different glucose concentrations. #P<0.05 stimulated *vs* non stimulated. **(D)** 6 hours with different glucose concentrations. *P<0.05 D-BMDM 25Mm+ *vs* ND-BMDM 25mM-. **(E)** 24 hours with different glucose concentrations. +P<0.05 5.5mM+ *vs* 25mM+. IL-6 measurement was performed by enzyme-linked immunosorbent assay. Results represent mean ± SEM. N = 7-13. BMDM: Bone-Marrow Derived Macrophages, from diabetic (D-) and non-diabetic (ND-) C57BL/6 mice.

We have also investigated the effect of hyperglycemia concomitant with LPS+Nigericin stimulation to assess the secretion of the pro-inflammatory cytokine IL-6 (**Figure 4**). After 2, 4, 6 and 24 hours of incubation, BMDM from diabetic and non-diabetic animals released a more significant amount of IL-6 at concentrations of 5.5mM and 25 mM of glucose, with LPS+Nigericin stimulation (**Figures 4A–E**). At 6 hours, we observed that D-BMDM increased the secretion of this cytokine in hyperglycemic medium with stimulation (**Figure 4D**). After 24 hours, ND-BMDM and D-BMDM in hyperglycemic medium with stimulation, secreted more this cytokine when compared with macrophages cultured in norgmoglycemic medium with LPS+Nigericin (**Figure 4E**).

### 3.4 TNF-α cytokine secretion in BMDM of diabetic animals stimulated with LPS + Nigericin

**Figure 5:**
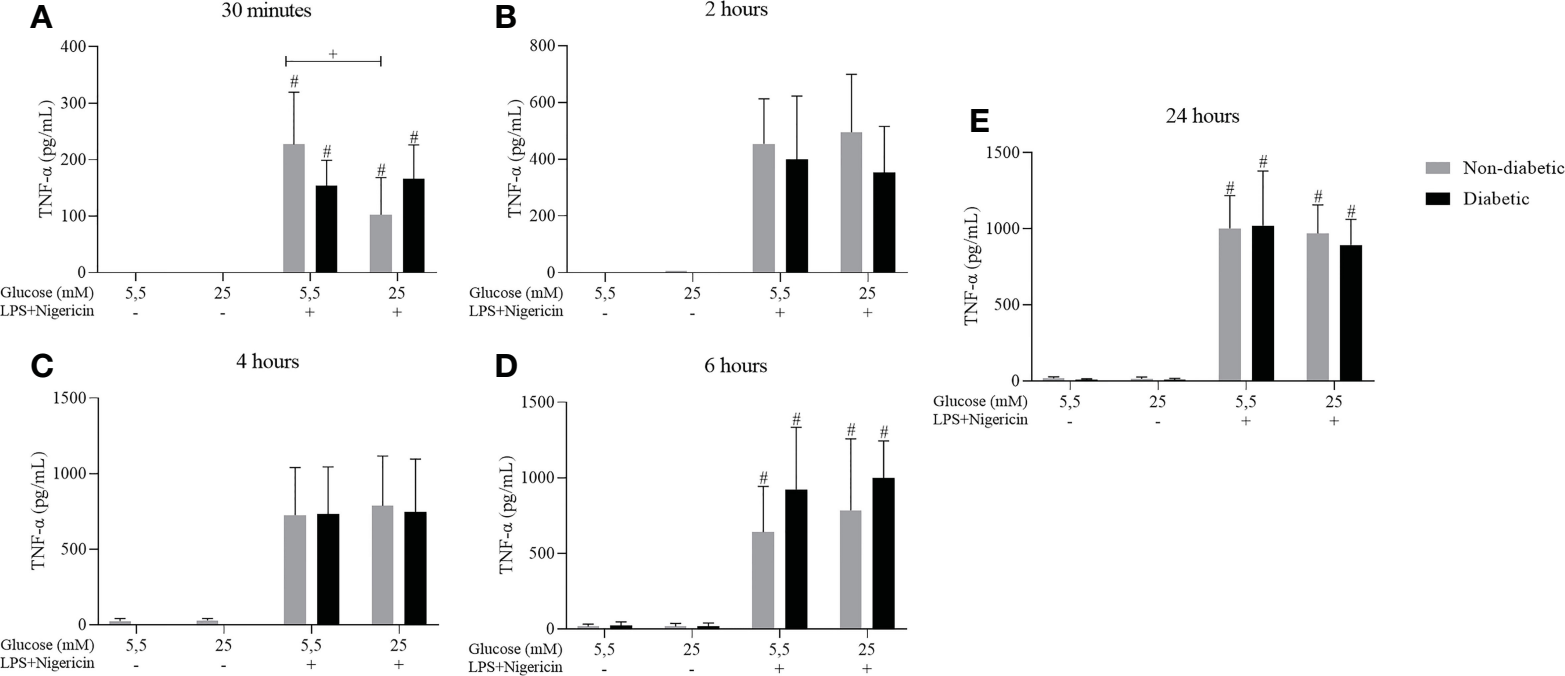
TNF-α secretion by ND-BMDM and D-BMDM with Nigericin priming and LPS stimulation. IL-6 release after **(A)** 30 minutes with different glucose concentrations. #P<0.05 stimulated *vs* non stimulated. + P<0.05 D-BMDM 25Mm+ *vs* ND-BMDM 25mM-. **(B)** 2 hours with different glucose concentrations. **(C)** 4 hours with different glucose concentrations. **(D)** 6 hours with different glucose concentrations. #P<0.05 stimulated *vs* non stimulated **(E)** 24 hours with different glucose concentrations. #P<0.05 stimulated *vs* non stimulated. TNF-α measurement was performed by enzyme-linked immunosorbent assay. Results represent mean ± SEM. N = 7-13. BMDM: Bone-Marrow Derived Macrophages, from diabetic (D-) and non-diabetic (ND-) C57BL/6 mice.

Cytokine measurements were performed to measure the secretion of TNF-α. After 30 minutes, 2, 4, 6, and 24 hours of incubation, BMDM from diabetic and non-diabetic animals released higher levels of TNF-α at concentrations of 5.5 and 25 mM glucose, with stimulation of LPS+Nigericin (**Figure 5**).

### 3.5 IL-1β cytokine secretion in BMDM of diabetic animals stimulated with LPS + Nigericin

**Figure 6:**
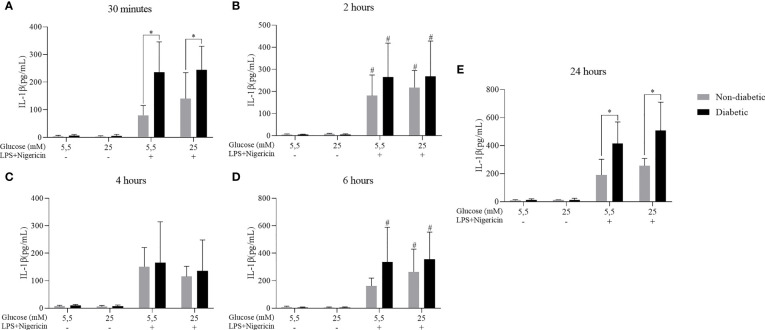
IL-1β secretion by ND-BMDM and D-BMDM with Nigericin priming and LPS stimulation. IL-1β release after **(A)** 30 minutes with different glucose concentrations. *P<0.05 D-BMDM 5.5Mm+ *vs* ND-BMDM 5.5mM+. *P<0.05 D-BMDM 25Mm+ *vs* ND-BMDM 25mM+. **(B)** 2 hours with different glucose concentrations. #P<0.05 stimulated groups *vs* non stimulated. **(C)** 4 hours with different glucose concentrations. **(D)** 6 hours with different glucose concentrations. #P<0.05 stimulated groups *vs* non stimulated. **(E)** 24 hours with different glucose concentrations. *P<0.05 D-BMDM 5.5Mm+ *vs* ND-BMDM 5.5mM+. *P<0.05 D-BMDM 25Mm+ *vs* ND-BMDM 25mM+. IL-1β measurement was performed by enzyme-linked immunosorbent assay. Results represent mean ± SEM. N = 7-13. BMDM: Bone-Marrow Derived Macrophages, from diabetic (D-) and non-diabetic (ND-) C57BL/6 mice.

The secretion of the IL-1β cytokine against the effect of hyperglycemia concomitant with the stimulation by LPS+Nigericin was also evaluated. It was observed that after 30 minutes, 2, 4, 6 and 24 hours of incubation, BMDM from diabetic and non-diabetic animals released a more significant amount of IL-1β at concentrations of 5.5 and 25 mM glucose, with LPS+Nigericin stimulation (**Figure 6**). At 30 minutes, D-BMDM with stimulation secreted IL-1β more than ND-BMDM under the same conditions (**Figure 6A**). At 4 and 24 hours, D-BMDM in hyperglycemic medium with stimulation had an increase in the secretion of this cytokine when compared with ND-BMDM in the same condition (**Figures 6C** and **E**). We also assessed Nitric Oxide and IL-10 levels, but no significant differences were observed (**Supplementary Figures 1** and **2**).

## 4 Discussion

It is already described that patients can establish a chronic inflammation state under diabetic conditions, characterized by a decompensated secretion of pro-inflammatory cytokines, such as TNF-α, IL-1β and IL-6, and it is suggested as the major cause of comorbidities related to diabetes (33). Furthermore, it has been identified that peritoneal macrophages, when exposed to high levels of glucose secrete greater amounts of TNF-α, IL-1β, IL-6 and IL-12 in response to high glucose concentrations (34, 35). It has also been reported by Cheng et al. (36) that RAW264.7 cells significantly increase TNF-α production after being exposed to high glucose levels (36).

A study showed that, when cultivated in a hyperglycemic medium, BMDM secreted a more significant amount of TNF-α, but the expression of IL-6 under the same conditions was reduced (30). However, our studies observed that high glucose levels alone were not enough to stimulate the secretion of TNF-α, IL-1β and IL-6 by macrophages. On the other hand, when stimulated with LPS, there was a significant increase in the secretion of these cytokines. Furthermore, Tessaro et al. (37) reported that macrophages from different sites from diabetic animals have a dysregulated response when stimulated by LPS, causing the control of inflammation to be impaired (37). Our findings corroborate with previous studies where it was reported that high glucose concentrations (15mM, 25mM) do not alter the expression of TNF-α and IL-6 by macrophages, however, when stimulated with LPS, the secretion of these cytokines increase, showing that hyperglycemia it is not a sufficient stimulus for the high production of these cytokines by macrophages (35, 38).

High glucose conditions can cause mitochondrial dysfunction, increase the production of ROS and activates the autophagy pathway (29, 39–41). Furthermore, LC3b protein is involved in mitophagy (42), a process that removes dysfunctional mitochondria by the autophagic machinery (43). Therefore, we suggest that the high levels of LC3b and beclin-1 expression by ND-BMDM, when compared to D-BMDM identified in this study are due to the normal cellular regulation process in response to stress caused by the hyperglycemic condition, suggesting that the autophagy pathway is impaired in macrophages from diabetic animals, which reinforces that these cells are sensitized when exposed to the hyperglycemic state *in vivo* (44–47). Furthermore, we observed a reduction in LC3b expression by ND-BMDMs and D-BMDMs stimulated and in hyperglycemic conditions. Our findings corroborate with study that showed a decrease in LC3b expression in THP-1-derived macrophages exposed to high concentrations of glucose with the inflammasome pathway activated (48). However, in this study performed by Dai et al. (48), THP-1-derived macrophages with the inflammasome pathway activated were exposed to a long-term hyperglycemic condition for 2 and 3 days (48). In our study, LPS+Nigericin stimulated BMDM were exposed to high glucose concentration for 30 minutes, which suggests that hyperglycemia impairs the autophagy process even in short-term condition.

It was already described that alterations in the autophagy pathway can directly interfere in the inflammatory response of diabetic individuals, making them susceptible to the development of infections (12) (49, 50). Combined with that, nigericin is known to be an inducer of the NLRP3 inflammasome pathway, where the formation of this complex results in the production of IL-1β (51). In our studies, we observed that macrophages from diabetic animals, when stimulated, secreted a greater amount of the cytokine IL-1β, showing that there is an exacerbated production of this cytokine when stimulated by LPS. Since the relationship between the autophagy process and IL-1β cytokine secretion has been widely studied, it has been reported that this process is responsible for sequestering this cytokine and preventing its secretion (52), and a negative regulation of this pathway can lead to an increase of IL-1β release (21, 53). These results suggest that, besides macrophages of diabetic animals being previously sensitized by hyperglycemia, the failure of the autophagy machinery may be contributing to the decompensated secretion of this cytokine.

With the changes observed in our study, we can observe that hyperglycemia plays an essential role in the inflammatory response of BMDMs from diabetic mice, since the high concentration of glucose with LPS stimulation led to significant changes in the secretion of inflammatory mediators and in the autophagy process, having a direct effect on cellular homeostasis.

## 5 Conclusion

The high concentration of glucose alters the inflammatory pathways in macrophages after LPS stimulation, disrupting the secretion of pro-inflammatory cytokines by these cells, leading to an impaired inflammatory response against infections. Furthermore, we observed that the sensitization caused by hyperglycemia in macrophages can downregulate the expression of proteins involved in the autophagy pathway, impairing cellular homeostasis, suggesting the main role of this mechanism over macrophages under diabetic conditions.

## Data availability statement

The raw data supporting the conclusions of this article will be made available by the authors, without undue reservation.

## Ethics statement

This study was accepted by the Ethics Committee on Animal Use (CEUA) at the School of Pharmaceutical Sciences (FCF), the University of São Paulo, Brazil (protocol number: CEUA/FCF/570), and all experiments were conducted in strict accordance with the principles and guidelines of the National Council for the Control of Animal Experimentation (CONCEA).

## Author contributions

ES and JM conceived and designed the experiments. ES, LQ, JG, KP and RB performed the experiments. ES, LQ, SE and JM analyzed the data. JM and SE contributed with reagents/materials/analysis tools. ES and JM wrote the paper with the assistance and contribution of all the authors. All authors contributed to the article and approved the submitted version.
