# Supplementary material for: The influence of high glucose conditions on macrophages and its effect on the autophagy pathway
Source: Front Immunol. 2023 Apr 12;14:1130662. doi: 10.3389/fimmu.2023.1130662 (PMC10130370; doi:10.3389/fimmu.2023.1130662)
Supplement: Supplementary file 1 [file DataSheet_1.pdf]

Supplementary Material

The Influence of High Glucose Conditions on Macrophages and Its Effect on the Autophagy Pathway

Emanuella S. A. Sousa[1], Luiz A. D. Queiroz[1], João P. T. Guimarães[1], Kamilla C. Pantoja[1], Rafael S. Barros[1], Sabrina Epiphanio[2], Joilson O. Martins[1*]

[1] Laboratory of Immunoendocrinology, School of Pharmaceutical Sciences, Department of Clinical and Toxicological Analyses, University of São Paulo, São Paulo, SP, Brazil.

[2] Laboratory of Malaria Cellular and Molecular Immunopathology, School of Pharmaceutical Sciences, Department of Clinical and Toxicological Analyses, University of São Paulo, São Paulo, SP, Brazil.

*** Corresponding authors**

Joilson O. Martins, PhD
Laboratory of Immunoendocrinology
Department of Clinical and Toxicological Analyses
School of Pharmaceutical Sciences
University of São Paulo, Sao Paulo, Brazil
Av. Prof. Lineu Prestes 580 - Bloco 17
05508-000, São Paulo, SP, Brazil
Phone 55-11-3091-3662
FAX 55-11-3813-2197
e-mail: martinsj@usp.br

Keywords: Inflammation, Diabetes *mellitus*, Hyperglycemia, Autophagy

1. Supplementary Figure

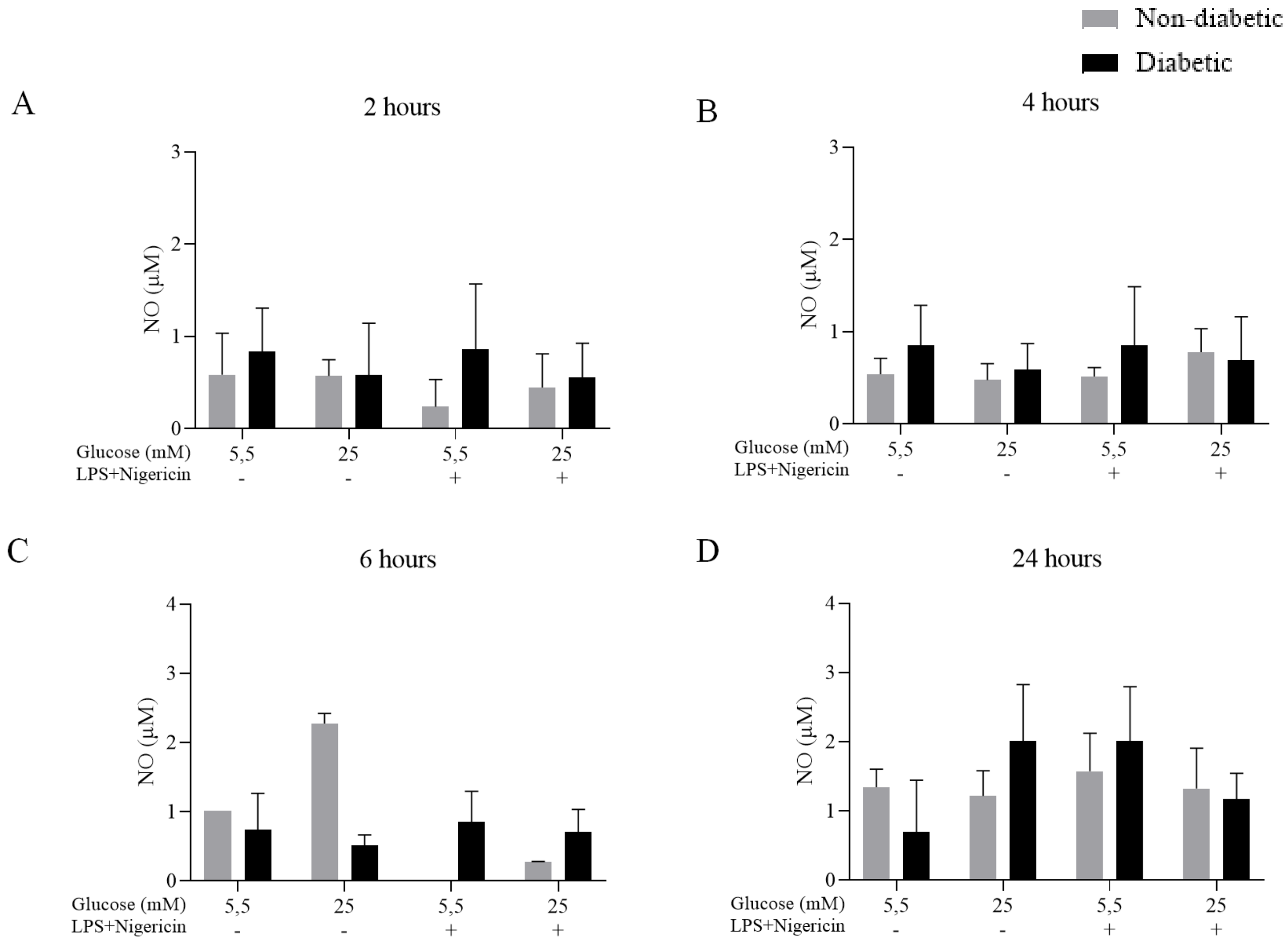

Supplementary Figure 1. Nitric Oxide (NO) secretion by ND-BMDM and D-BMDM with Nigericin priming and LPS stimulation. Release of nitric oxide after (A) 2 hours with different glucose concentrations. (B) 4 hours with different glucose concentrations. (C) 6 hours with different glucose concentrations. (D) 24 hours with different glucose concentrations. Nitric oxide dosage was performed by Griess reaction. Results represent mean ± SEM. N = 6-8.

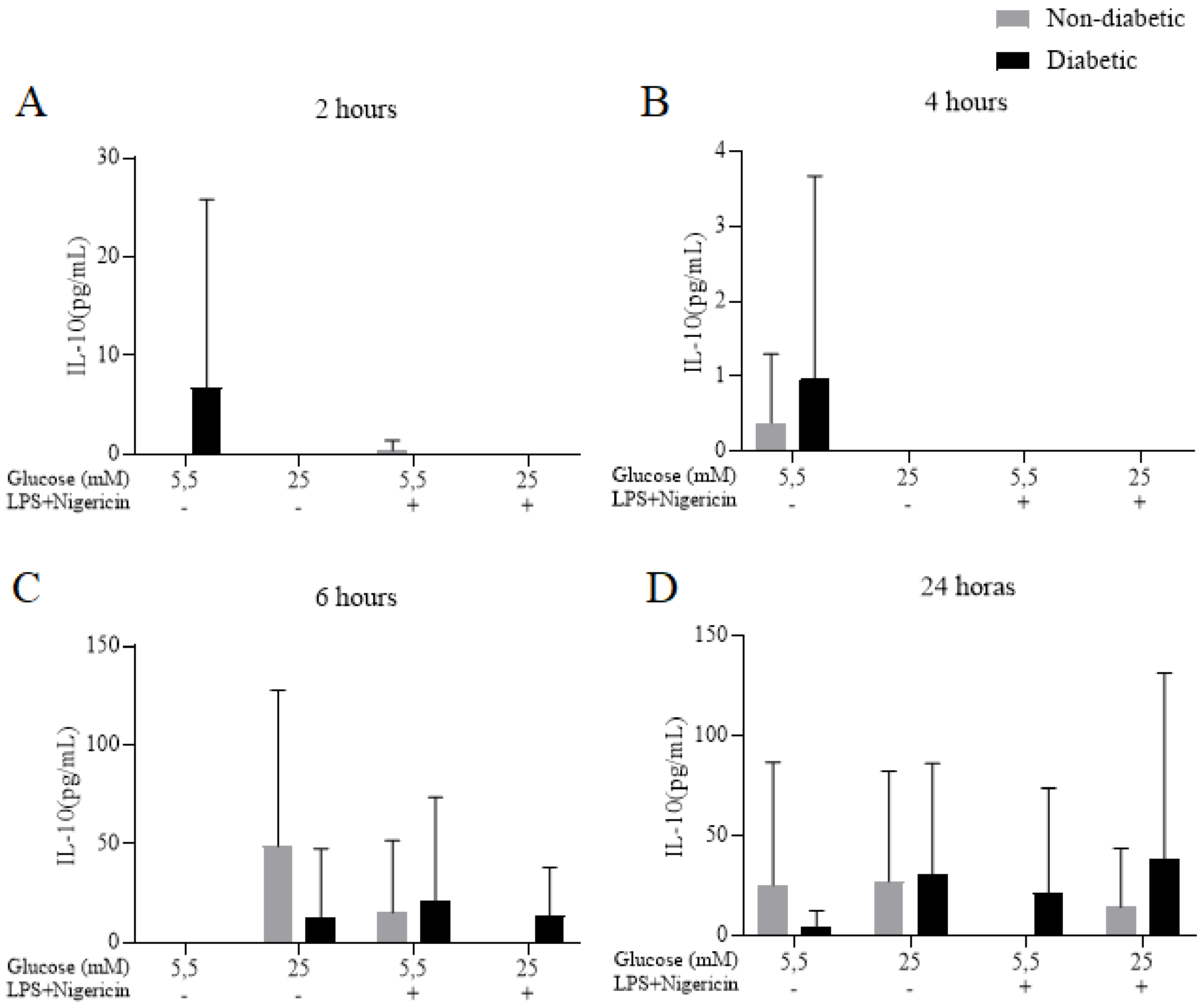

Supplementary Figure 2. IL-10 secretion by ND-BMDM and D-BMDM with Nigericin priming and LPS stimulation. Release of IL-10 after (A) 2 hours with different glucose concentrations. (B) 4 hours with different glucose concentrations. (C) 6 hours with different glucose concentrations. (D) 24 hours with different glucose concentrations. IL-10 measurement was performed by enzyme-linked immunosorbent assay. Results represent mean ± SEM. N = 6-8.

Supplementary Figure 3. Western blot membranes probed with anti-mouse Beclin1, anti-mouse LC3b and anti-mouse β-actin.

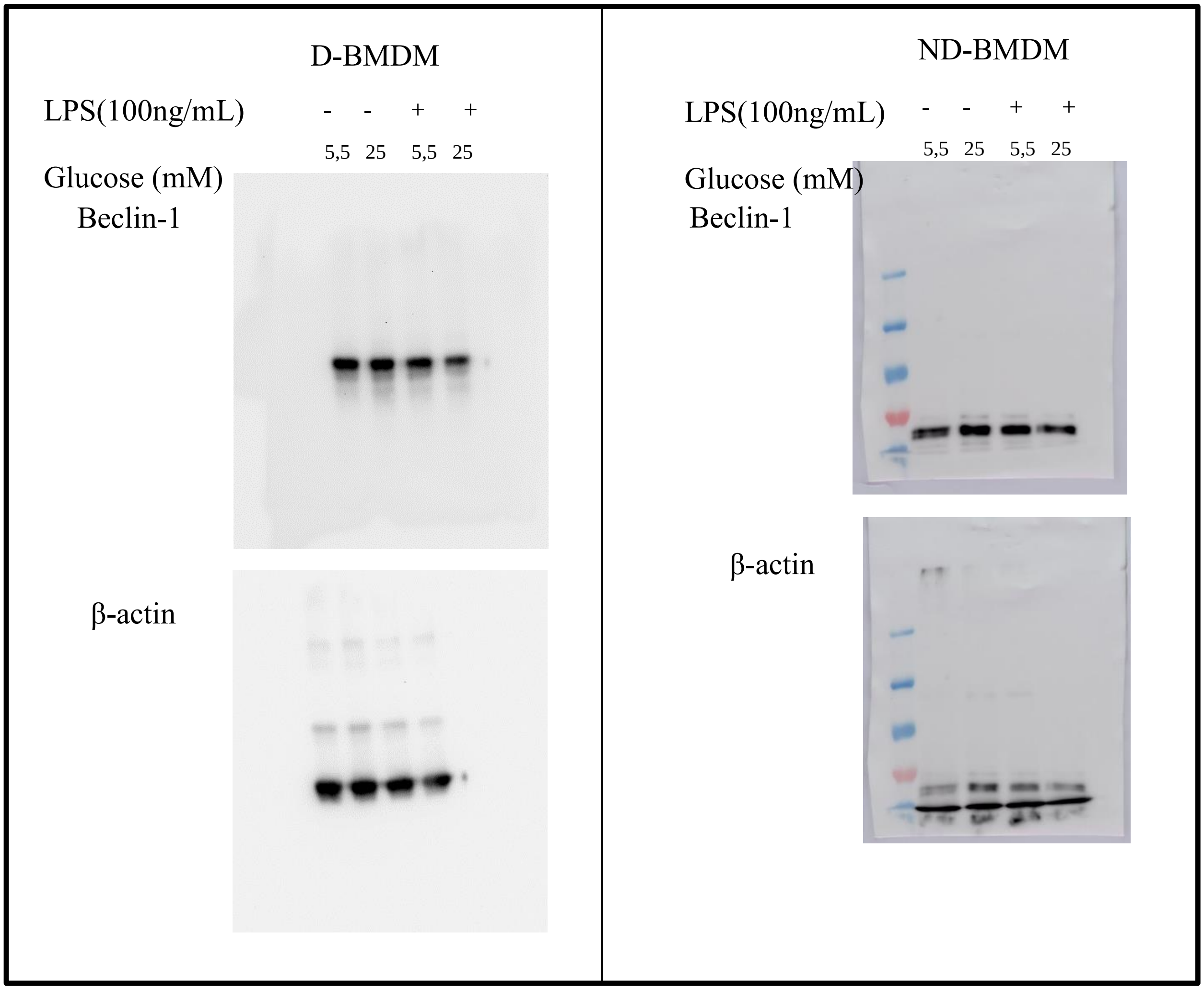

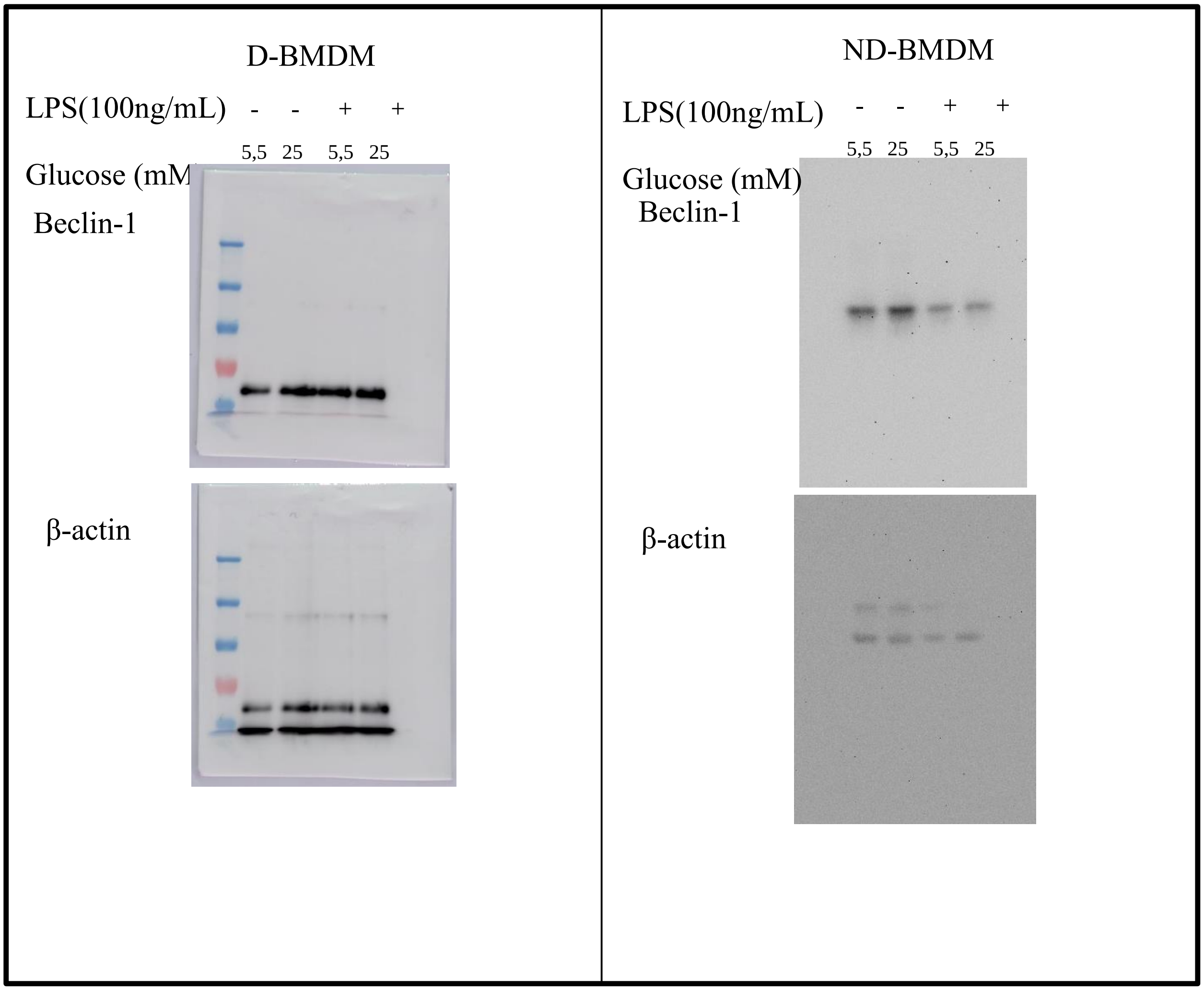
D-BMDM
LPS(100ng/mL) - - + +
5,5 25 5,5 25
Glucose (mM)
Beclin-1
β-actin
ND-BMDM
LPS(100ng/mL) - - + +
5,5 25 5,5 25
Glucose (mM)
Beclin-1
β-actin

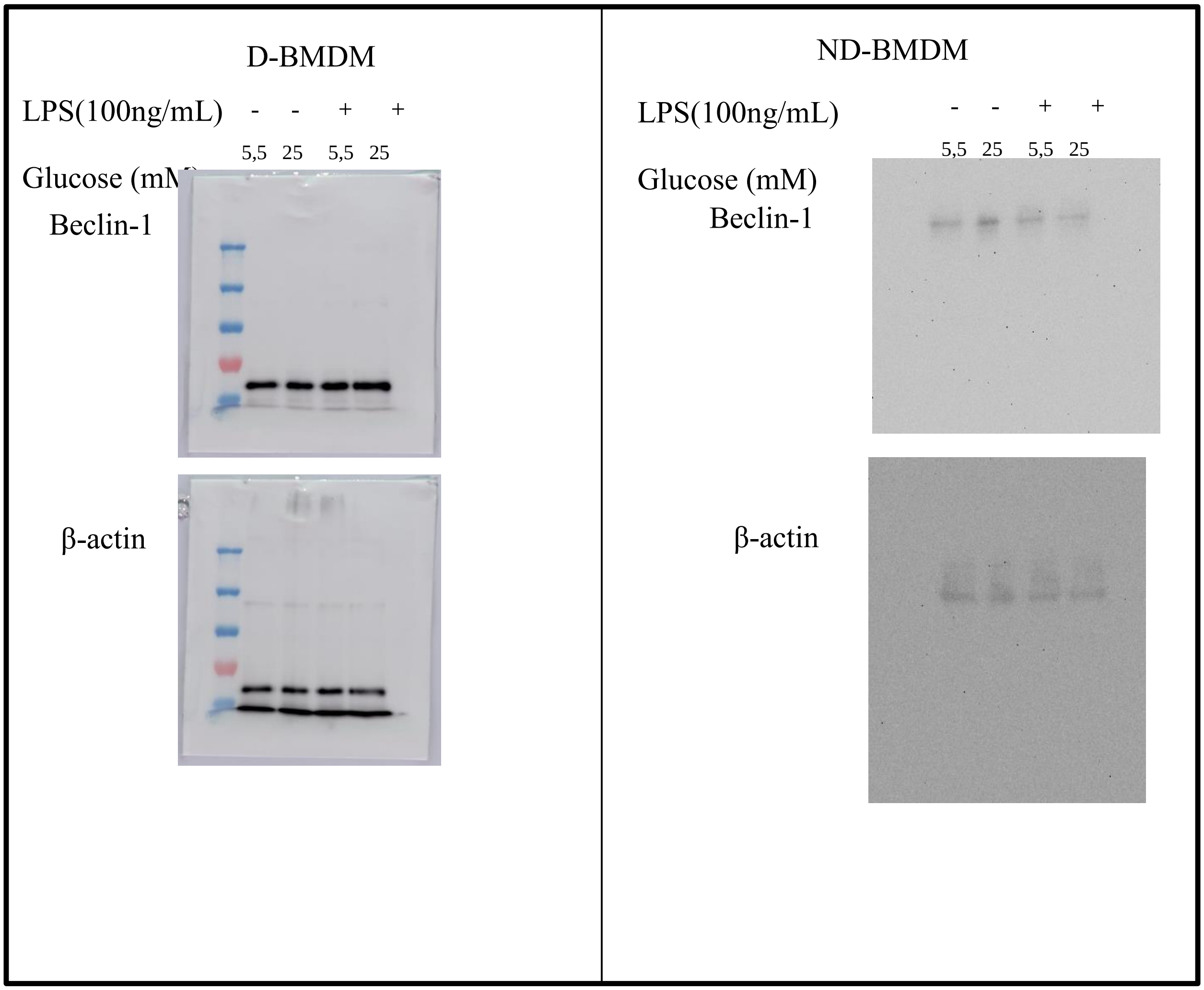
D-BMDM
LPS(100ng/mL) - - + +
5,5 25 5,5 25
Glucose (mM)
Beclin-1
β-actin
ND-BMDM
LPS(100ng/mL) - - + +
5,5 25 5,5 25
Glucose (mM)
Beclin-1
β-actin

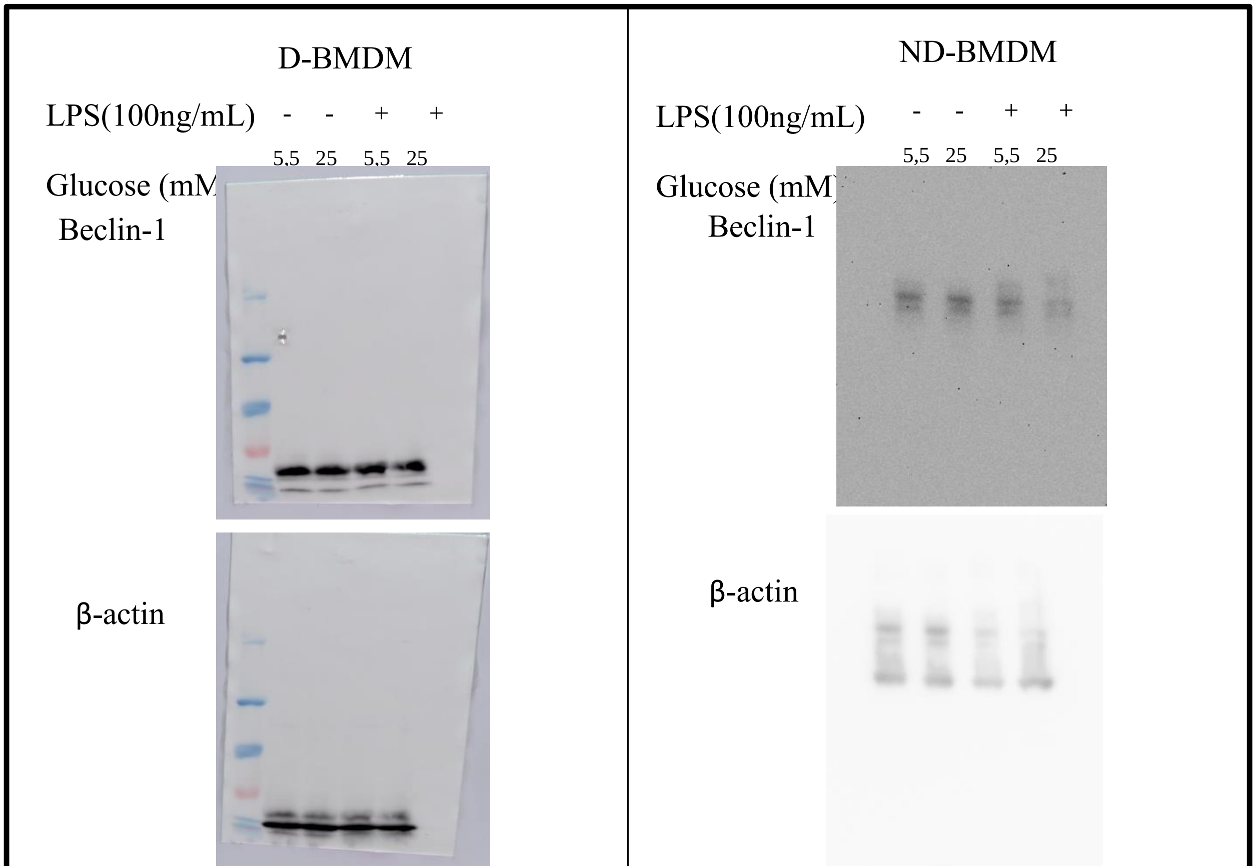
D-BMDM
LPS(100ng/mL) - - + +
5,5 25 5,5 25
Glucose (mM)
Beclin-1
β-actin
ND-BMDM
LPS(100ng/mL) - - + +
5,5 25 5,5 25
Glucose (mM)
Beclin-1
β-actin

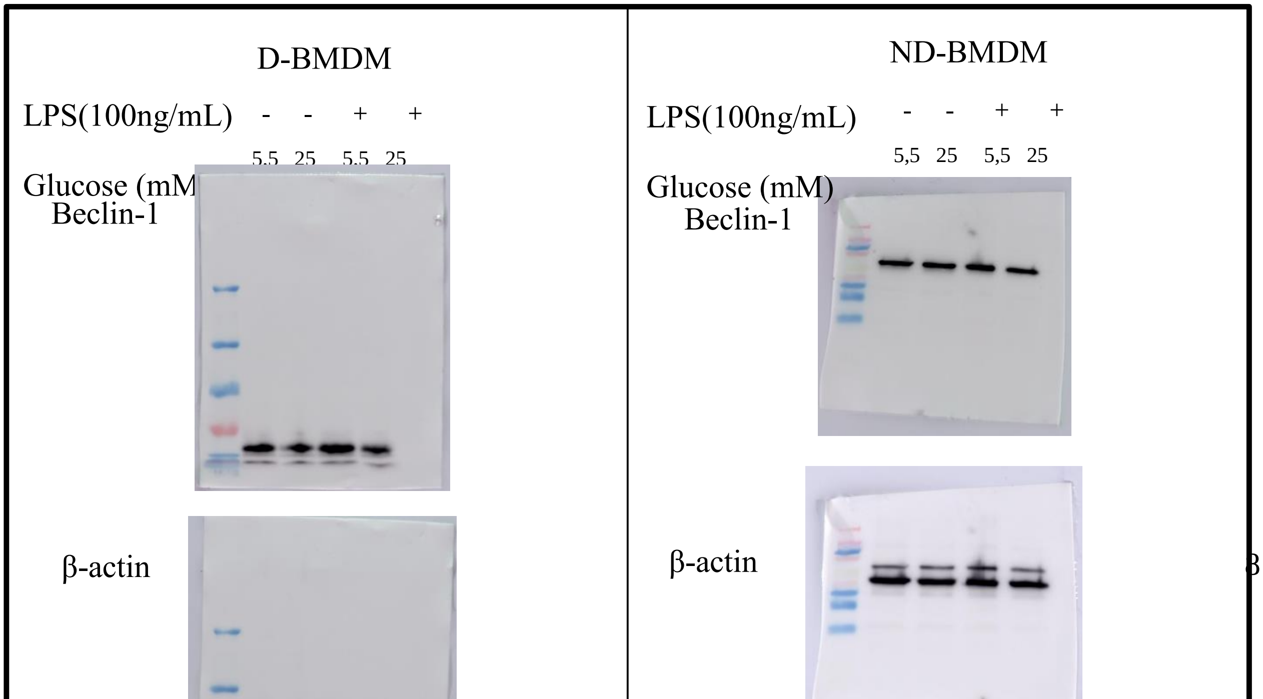
D-BMDM
LPS(100ng/mL) - - + +
5,5 25 5,5 25
Glucose (mM
Beclin-1
β-actin
ND-BMDM
LPS(100ng/mL) - - + +
5,5 25 5,5 25
Glucose (mM)
Beclin-1
β-actin
β

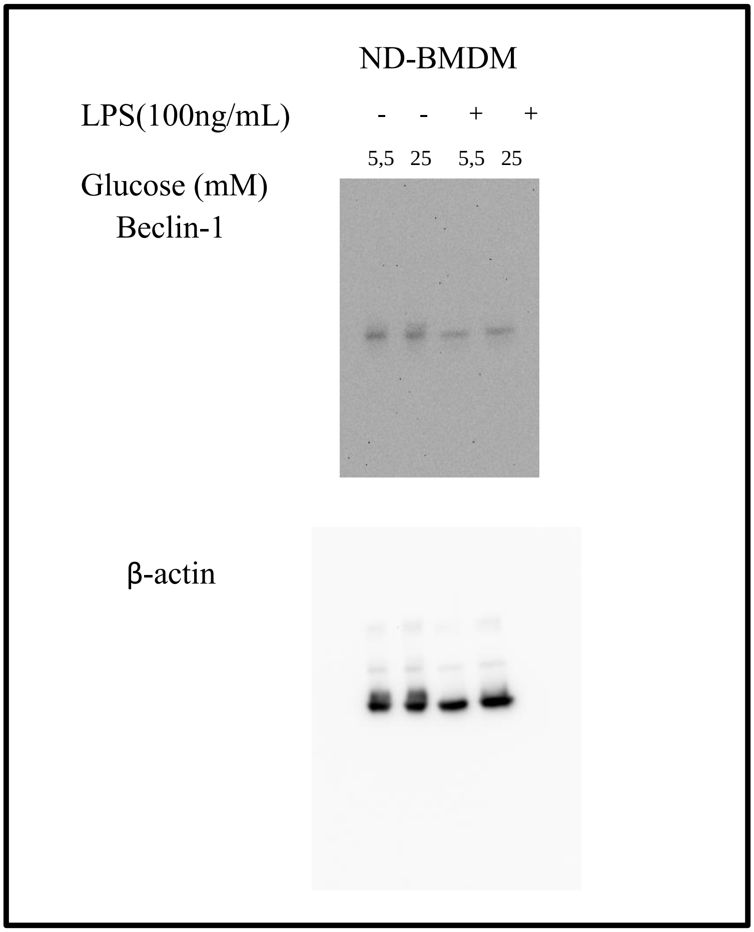
ND-BMDM
LPS(100ng/mL) - - + +
Glucose (mM) 5,5 25 5,5 25
Beclin-1
β-actin

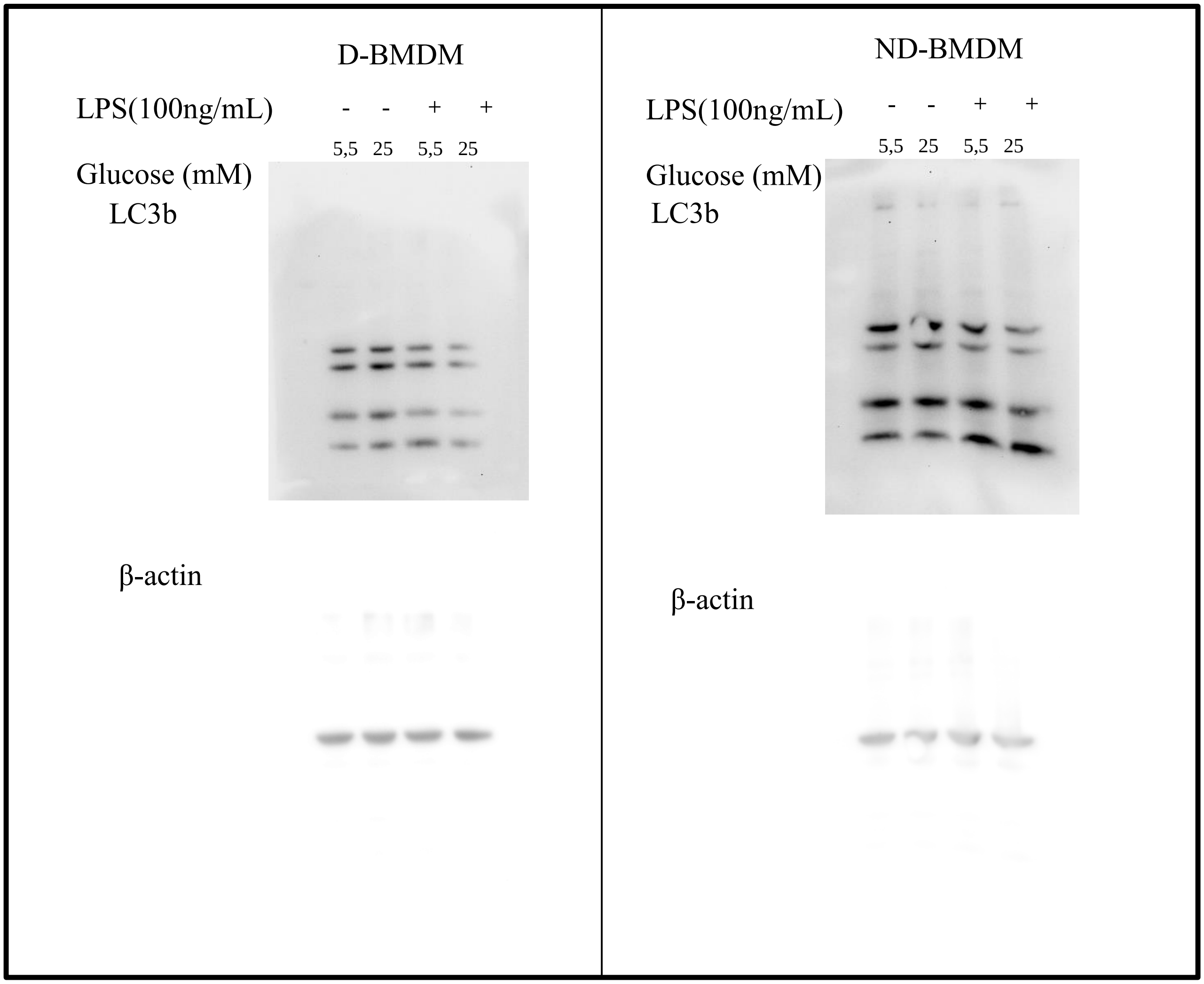
D-BMDM
LPS(100ng/mL) - - + +
5,5 25 5,5 25
Glucose (mM)
LC3b
β-actin
ND-BMDM
LPS(100ng/mL) - - + +
5,5 25 5,5 25
Glucose (mM)
LC3b
β-actin

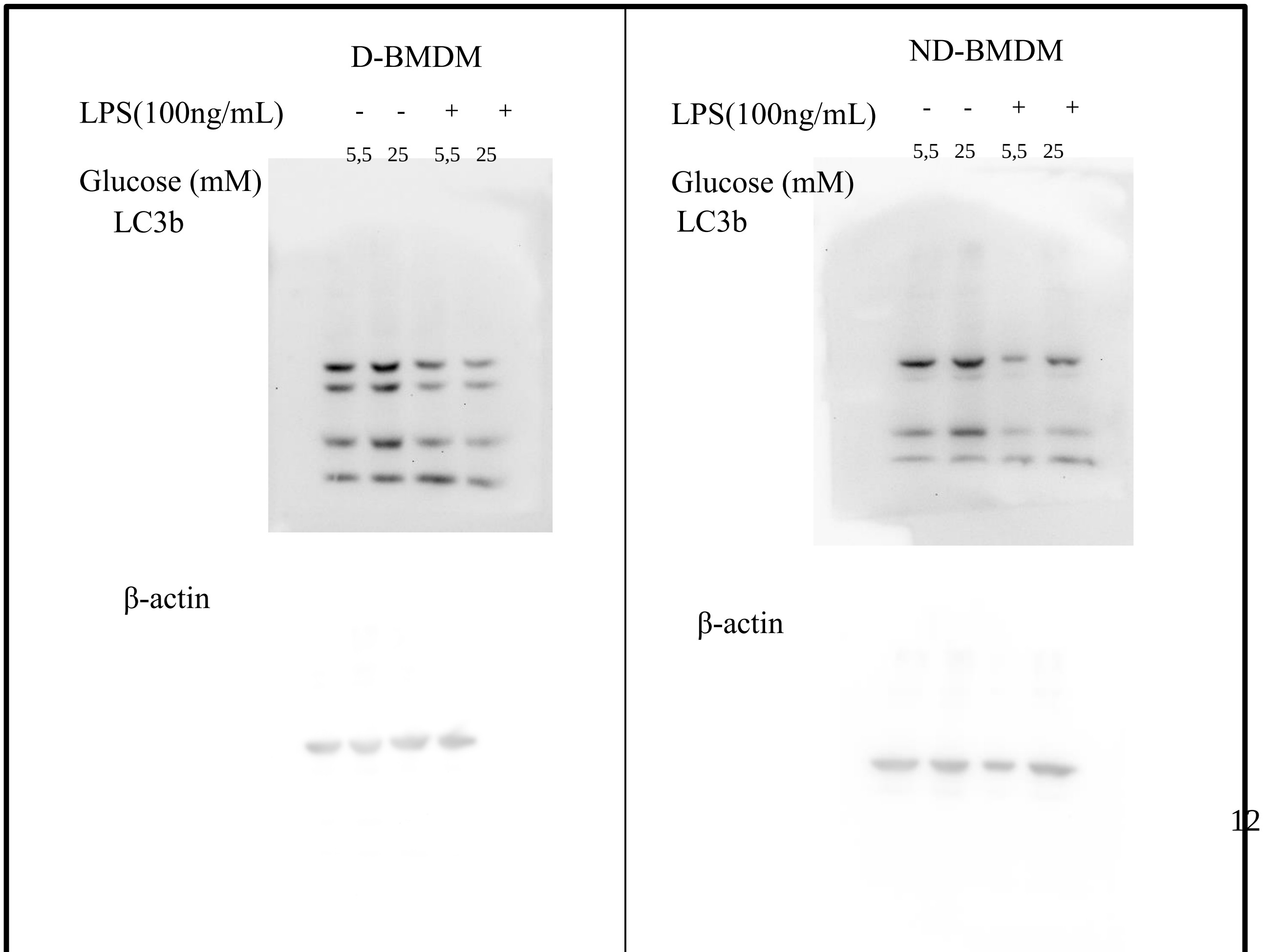
D-BMDM
LPS(100ng/mL) - - + +
Glucose (mM) 5,5 25 5,5 25
LC3b
β-actin
ND-BMDM
LPS(100ng/mL) - - + +
Glucose (mM) 5,5 25 5,5 25
LC3b
β-actin

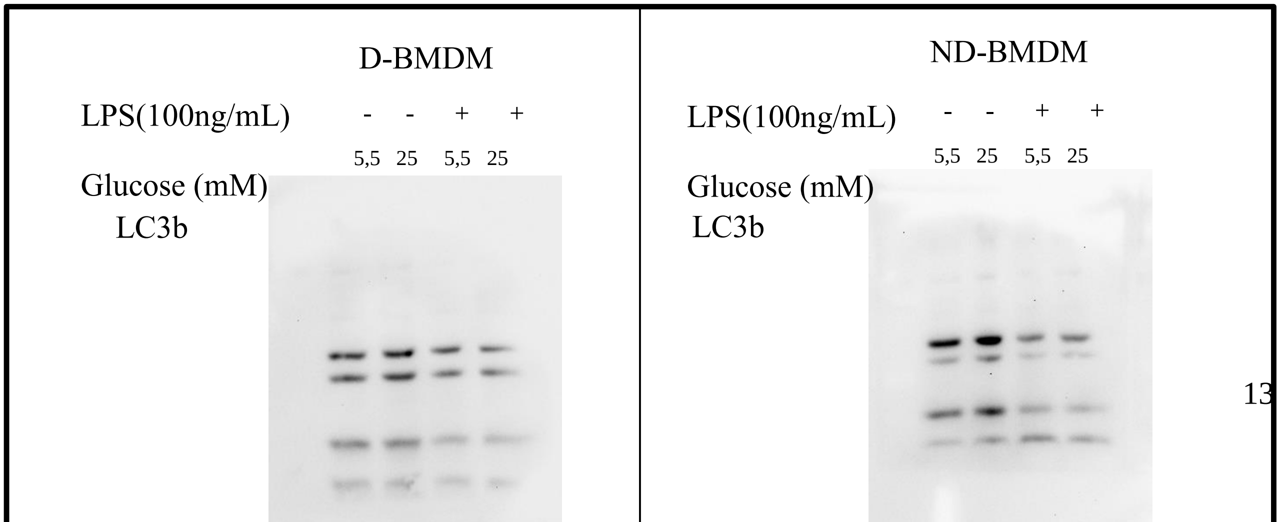
D-BMDM
LPS(100ng/mL) - - + +
5,5 25 5,5 25
Glucose (mM)
LC3b
ND-BMDM
LPS(100ng/mL) - - + +
5,5 25 5,5 25
Glucose (mM)
LC3b

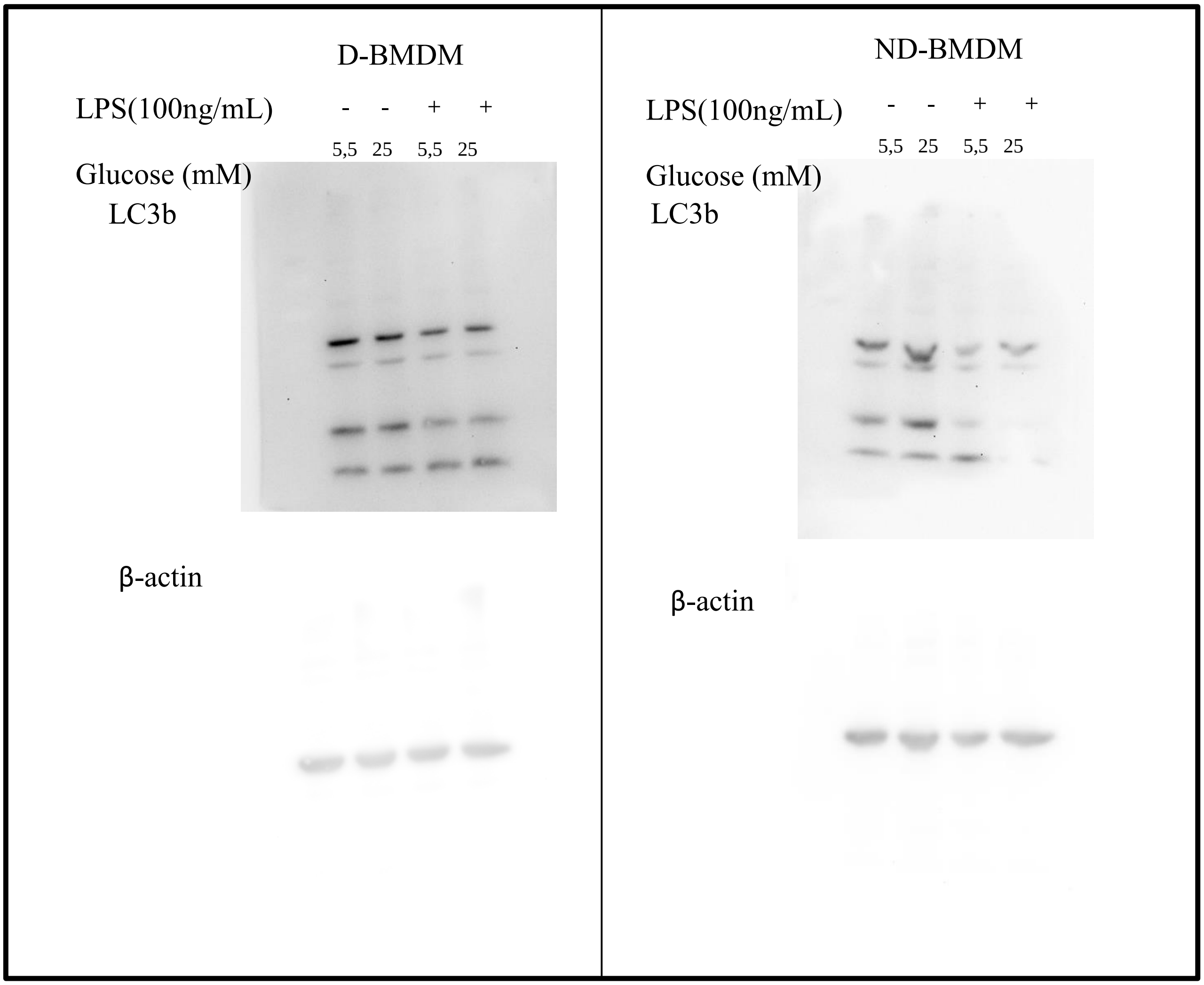
D-BMDM
LPS(100ng/mL) - - + +
Glucose (mM) 5,5 25 5,5 25
LC3b
β-actin
ND-BMDM
LPS(100ng/mL) - - + +
Glucose (mM) 5,5 25 5,5 25
LC3b
β-actin

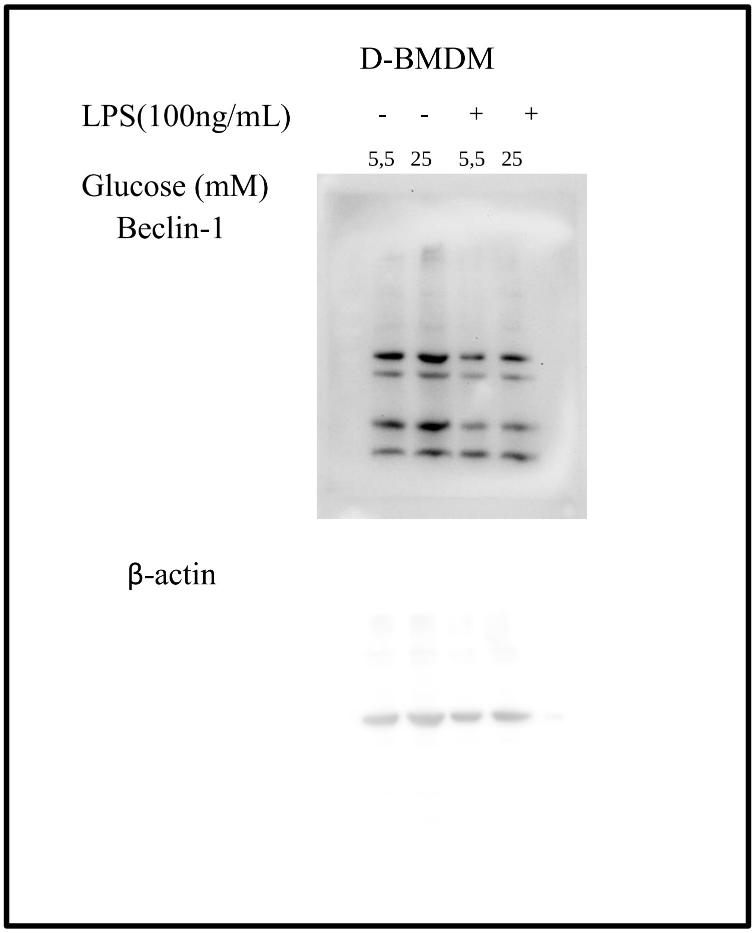
D-BMDM
LPS(100ng/mL) - - + +
5,5 25 5,5 25
Glucose (mM)
Beclin-1
β-actin
